# Unilateral Levator Aponeurosis Excision for Marcus Gunn Syndrome and Risk Factors of Residual Jaw Winking

**DOI:** 10.1155/2019/2058047

**Published:** 2019-11-04

**Authors:** Qingyao Ning, Jing Cao, Jiajun Xie, Qi Gao, Changjun Wang, Juan Ye

**Affiliations:** Department of Ophthalmology, The Second Affiliated Hospital of Zhejiang University, College of Medicine, Hangzhou, Zhejiang, China

## Abstract

**Purpose:**

To investigate the association of ptosis, levator, and jaw winking in Marcus Gunn jaw-winking synkinesis (MGJWS), and the risk factor of preservation and outcomes of the unilateral levator excision and frontalis suspension.

**Methods:**

Clinical features of MGJWS case series from 2011 to 2018 were retrospectively reviewed. Association between jaw winking and ptosis/levator function was statistically analyzed. The patients underwent unilateral levator excision and frontalis suspension using silicone rod or autogenous fascia lata. Clinical outcomes were evaluated in operated patients and the independent risk factors of residual jaw winking were investigated after a long follow-up.

**Results:**

There were 42 MGJWS patients in 2011 to 2018, accounting for 2.87% of all congenital blepharoptosis. 80% of mild jaw winking was accompanied with mild ptosis and fair levator function, and moderate-to-severe jaw winking was often accompanied with moderate-to-severe ptosis and poor levator function (*P* < 0.05). Ptosis showed a strong association with excursion of jaw winking (*R* = 0.785, *P* < 0.01). Jaw winking was resolved in all 34 operated patients with good correction of ptosis. Severity of jaw winking is an independent risk factor for the residual synkinesis after surgery. Severe preoperative jaw winking had an 18.05 times increased risk of postoperative residual synkinesis compared with moderate jaw winking (*P* < 0.05).

**Conclusions:**

In MGJWS eyelid excursion of jaw winking has a direct correlation with ptosis and dysfunction of levator muscle. Unilateral levator aponeurosis excision and frontalis suspension is an efficient approach for MGJWS. Severe jaw winking is a risk factor of residual eyelid synkinesis after surgery.

## 1. Introduction

Marcus Gunn syndrome, also known as Marcus Gunn jaw-winking synkinesis (MGJWS), was first described by Gunn in 1883 as a congenital unilateral ptosis with contraction of the levator palpebrae superioris in association with the external pterygoid muscle [1]. In previous studies, MGJWS was reported to occur in 2%–13% of patients with congenital ptosis [2–6]. Most cases are unilateral, although some bilateral cases and familial cases have been reported [7–12]. Infrequent cases with absence of ptosis in MGJWS were also reported in previous studies, which occurred in 1.2%–6.0% of MGJWS patients [13, 14]. The affected eyelid would elevate upward with external pterygoid movement such as opening of the mouth, jaw opening to the opposite side; with internal pterygoid movement such as jaw movement to the same side; and even with other variety of movements such as chewing, sucking, smiling, and so on [12, 15]. The underlying etiopathogenesis of the phenomenon has not been completely identified up to now. Most scientists believe that it involves the abnormal connection between the fifth cranial nerve and the third cranial nerve supplying the levator muscle at a central or peripheral level [16–20].

It is generally recommended by ophthalmologists that mild jaw winking (less than 2 mm) with mild ptosis could be clinically observed without surgical treatment if the patient did not consider it to be a cosmetic problem. For moderate-to-severe jaw winking, surgical management is considered to be the main therapeutic method. The aim of the operation is to eliminate synkinetic eyelid movement and correct ptosis. A variety of surgical techniques were reported, such as the Fasanella–Servat procedure [21], levator sling [22–24], modified levator resection or plication [25], and levator muscle excision followed by frontalis suspension [10, 12, 18, 26]. Of the above procedures, levator excision with bilateral frontalis suspension was a widely used technique for good results in both synkinesis elimination and bilateral symmetry [12, 27]. However, some patients were unwilling to get normal eyelids operated. In recent years, some ophthalmologists reported single eye surgery techniques, which got satisfied surgical outcomes as well.

The standard surgical procedure for MGJWS still remains controversial at present. The synkinesis is difficult to be completely eliminated with present surgical techniques. The association between clinical feature, surgical techniques, and prediction of surgery intervention was not well reported in previous studies. This work retrospectively collected congenital blepharoptosis patients over an 8-year period and studied all MGJWS cases with their clinical manifestations and outcomes of unilateral surgery, especially analyzed the association between eyelid synkinesis and blepharoptosis, and the risk factors of the residual jaw winking after surgery.

## 2. Materials and Methods

### 2.1. Patients and Investigations

We retrospectively reviewed patients with MGJWS within all congenital ptosis patients at the Eye Center of The Second Affiliated Hospital of Zhejiang University from January 2011 to December 2018. The study was approved by the institutional review board of the hospital and conducted in accordance with the Declaration of Helsinki. All patients who underwent surgical intervention had given their written informed consent before surgery. For the minors, the written informed consent forms were signed by their guardians. Signed consent for clinical photographs that permit identification of the patient was achieved.

Age, gender, affected eye, family history, previous surgical history, and other associated eye disorders (including strabismus, amblyopia, and eyelid entropion) were recorded. The following investigations were carefully assessed before surgical intervention: margin reflex distance (MRD) 1 of both eyes, levator function, jaw winking excursion of eyelid, and ocular protective mechanisms (Bell's phenomenon, orbicularis muscle function, corneal sensation, tear film break-up time, and Schirmer's test). Ptosis was recorded as mild (2 mm or less), moderate (2–4 mm), or severe (more than 4 mm). Levator function was recorded as good (more than 10 mm), fair (5–10 mm), or poor (<5 mm) [17]. Jaw wink was recorded as mild (less than 2 mm), moderate (2–5 mm), or severe (more than 5 mm) [12].

Patients with more than 2 mm ptosis and moderate-to-severe jaw winking were suggested to perform surgical intervention with unilateral levator aponeurosis excision and frontalis suspension of the affected eye. The ptosis correction results, symmetry of eyelids (including MRD1, eyelid counter, and symmetry of eyelid height), and postoperative complications were evaluated after surgery. To assess the result of obliteration of jaw-winking phenomenon, resolution of jaw winking was defined as 1 mm or less excursion of affected upper eyelid with synkinetic mouth movement; improvement was defined as a decrement of 2 mm or more, but with more than 1 mm remaining excursion of affected upper eyelid with synkinesis; if the excursion decreased less than 2 mm and with more than 1 mm remaining excursion, it was considered as no improvement [12]. Patients were examined at 2 week and 1, 3, 6, and 12 months, and then every year after operation. The patients of moderate-to-severe jaw winking who underwent surgical procedure and had more than 6 months follow-up would be further analyzed for evaluation of surgical management.

### 2.2. Surgical Methods

Surgery was performed under general anesthesia in patients younger than 18 years old. Adult patients underwent surgeries under local anesthesia. All the patients underwent unilateral levator muscle excision followed by frontalis suspension of the involved eye. In the patients who were older than 4 years old, autogenous fascia lata was used as the suspension material. In patients younger than 4 years old for whom the autogenous fascia lata was hard to harvest, and in patients who were deficient of ocular protection, silicone rod was used as the suspension material instead of autogenous fascia lata [28]. All surgical procedures were performed by one senior oculoplastic surgeon (JY).

**Figure 1:**
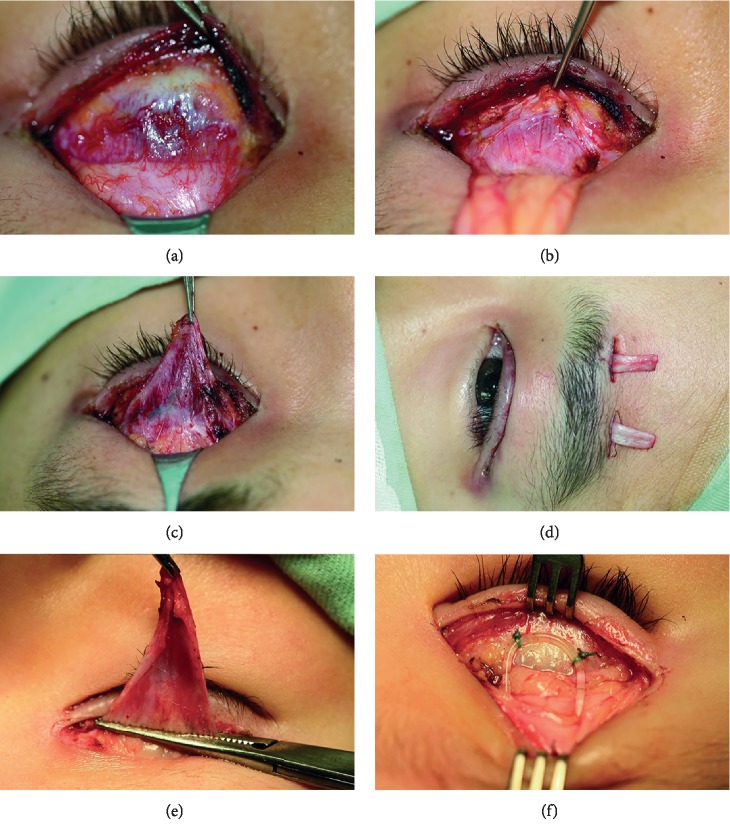
Unilateral levator excision and frontalis suspension for MGJWS. (a–d) Procedures of levator excision and frontalis suspension with autogenous fascia lata. (a) Exposure of Müller muscle and levator aponeurosis. (b, c) The levator aponeurosis is transected from tarsal plate and Müller muscle, and dissected upward to Whitnall's ligament. (d) Frontalis suspension with two pieces of autogenous fascia lata. (e, f) Procedures of silicone rod suspension of a 2-year-old child with right-side MGJWS. (e) Levator muscle was dissected to Whitnall's ligament and held with a hemostat before transection. (f) The silicone rod was fixed on the upper 1/3 of the tarsal plate and the other two ends going through the orbital septum tunnel were fixed on the frontalis.

Eyelid skin crease incision was used. Müller's muscle and levator aponeurosis were exposed through standard procedure. Levator aponeurosis was transected horizontally from tarsal plate and Müller's muscle. The medial and lateral edges of the levator aponeurosis were carefully incised as completely as possible. And then, the levator aponeurosis was dissected up to 1-2 mm under the Whitnall's ligament, and excised after being held with a hemostat. Bipolar cautery was applied to cauterize the stump of the muscle and the vertical lateral edges. Autogenous fascia lata was harvested with Crawford's technique [29]. Frontalis suspension was performed with the method described by Yoon and Lee [30]. Eyelid height was set at the estimated position according to both MRD1 and the height of contralateral normal eyelid (Figures 1(a)–1(d)). For the young patients who underwent silicone rod suspension, the middle part of the silicone rod was fixed on the tarsal plate with 5–0 nonabsorbable suture. The two ends of silicone rod were passed through the lateral and medial orbital septum tunnels, respectively, and then formed a triangular frontalis suspension and drawn out of the forehead incision (Figures 1(e) and 1(f)). The redundant fascia lata and silicone rod were cut off, and the skin incisions were sutured with 6–0 sutures. Artificial tears and ointment were applied to the eyes for corneal protection. Skin sutures were removed 2 weeks after operation.

### 2.3. Statistics

All statistical analyses were performed with SPSS software (V.20.0 for windows; SPSS, Chicago, Illinois, USA), and *P* < 0.05 was considered statistically significant. The Student's sample *t*-test was used for comparisons of continuous variables. For categorical variables, *χ*^2^ test were used. Association between jaw winking and ptosis or levator function was calculated with Pearson's correlation coefficients, and values >0.7 were regarded as “strong” correlation, and values between 0.50 and 0.70 were interpreted as “good” correlation. A binary multiple logistic regression analysis was performed to evaluate the independent association between potential risk factors and residual eyelid synkinesis with jaw winking. Associations between risk factors and residual eyelid synkinesis were estimated by the odds ratio (OR) and 95% confidence interval (CI). A value of *P* < 0.05 was considered statistically significant.

## 3. Results

From January 2011 to December 2018, a total of 1464 patients with congenial blepharoptosis were treated in the institution. Of these patients, 42 were diagnosed as MGJWS, accounting for 2.87% of all congenital blepharoptosis. Patients' age ranged from 2–48 years, with a mean age of 11.7 years. Twenty (47.6%) patients were male and 22 (52.4%) were female. In all 42 patients, jaw-winking synkinesis with ptosis was found involving the unilateral eye (right eye involved in 15 (35.7%) patients, and left eye involved in 27 (64.3%) patients) without any familial history or other congenital diseases. Six (14.3%) patients had ipsilateral amblyopia. Three (7.1%) patients had strabismus, of whom 1 had ipsilateral vertical strabismus, 1 had vertical strabismus of the unaffected eye, and 1 had horizontal strabismus; all the 3 patients underwent strabismus surgery before ptosis correction. No patients had undergone surgery intervention for ptosis in other medical institutions before. One adult patient (44 years old) had limitation of upward eye movement with absence of Bell's phenomenon. Therefore, the patient underwent frontalis suspension using silicone rod instead of autogenous fascia lata. All the other patients showed normal eye movement and good ocular protective mechanisms.

**Figure 2:**
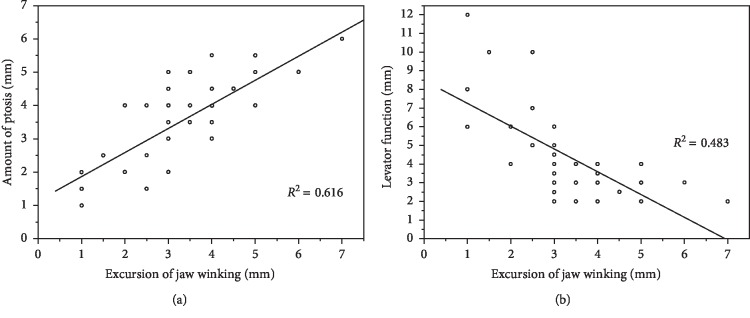
Association between ptosis/levator function and jaw winking in 42 MGJWS patients analyzed by a linear regression. Scatter plots (a) show a significant direct relationship between ptosis and jaw winking (*R* = 0.785, *P* < 0.01). Scatter plots (b) show a significant inverse relationship between levator function and jaw winking (*R* = −0.695, *P* < 0.01).

The mean palpebral fissure height of the affected eye was 4.40 mm (range: 1.5∼7.0 mm) and that of the normal eye was 7.99 mm (range: 7.0∼9.0 mm). Mean MRD1 of the ptotic eye was −0.57 mm (range: −3.5∼2.0 mm), with 7 (16.7%) mild ptosis patients, 24 (57.1%) moderate ptosis, and 11 (26.2%) severe ptosis. The mean levator function of the affected eyes was 4.37 mm (range: 2.0–12.0 mm), of 3 (7.1%) patients was good, 11 (26.2%) was fair, and 28 (66.7%) was poor. The mean excursion of jaw winking in primary gaze was 3.36 mm (range: 1.0–7.0 mm), with 5 (11.9%) eyelids showing mild jaw winking, 29 (69.1%) showing moderate jaw winking, and 8 (19.0%) showing severe jaw winking. The correlation between the classification of jaw winking and ptosis or levator function is shown in Table 1. Most of mild jaw winking was accompanied with mild ptosis (80%, *P* ≤ 0.001) and fair levator function (60%, *P*=0.002 < 0.01). However, moderate-to-severe jaw winking often showed a moderate-to-severe ptosis and poor levator function. Of these, 69% of moderate jaw winking was accompanied by moderate ptosis, and 65.2% of severe jaw winking was accompanied by severe ptosis (*P*=0.037 < 0.05). Pearson's correlation coefficient between the excursion of jaw winking and ptosis was 0.785 (*P* ≤ 0.001) indicating a strong direct relationship, and between the excursion of jaw winking and levator muscle function was −0.695 (*P* ≤ 0.001) indicating a good correlation (Figure 2).

Thirty-four patients with moderate-to-severe MGJWS underwent surgery intervention and had more than 6 months follow-up. Five patients with mild jaw winking (1 mm excursion in 4 patients and 1.5 mm in 1 patient) were suggested to have further clinical observation, since the patients or their parents did not consider jaw winking and ptosis problematic enough to require surgery intervention immediately. One patient with severe jaw winking and severe ptosis had undergone surgical treatment, but was not included in the analysis due to insufficient follow-up period. Two patients with moderate jaw winking and moderate ptosis were waiting for operation.

In the 34 operated patients, there were 27 with moderate jaw winking and 7 with severe jaw winking. The eyelid excursion of jaw winking was 3.69 ± 1.09 mm (range: 2.0–7.0 mm). Preoperative palpebral fissure height of the unaffected eyes was 7.97 ± 0.49 mm (range: 7.0–9.0 mm) and that of the affected eyes was 4.15 ± 1.17 mm (range: 1.5–6.5 mm) (*P*=0.009 < 0.01). MRD1 of the affected eyes ranged from −3.5 mm to 1.5 mm. Of these patients, 30 patients underwent levator aponeurosis excision of affected eye and unilateral frontalis suspension with autogenous fascia lata, and 4 patients underwent levator aponeurosis excision of affected eye and unilateral frontalis suspension with silicone rod. The mean follow-up period was 38.6 months (range: 6–72 months). At the end of follow-up, postoperative palpebral fissure height of the operated eye in primary gaze was 7.93 ± 0.58 mm (range: 6.0–9.0 mm), with no significant difference to the unaffected side (*P*=0.2497 > 0.05). The upper eyelid height in downgaze was 1-2 mm higher than normal side in 23 (67.7%) patients. The relative upper eyelid height to the normal side in downgaze was 0.82 ± 0.64 mm in all patients. Ptosis was functional corrected in all patients. MRD1 of the operated eye after surgery was 2.96 ± 0.48 mm (range: 1.5–4.0 mm). In 5 (14.7%) patients, postoperative MRD1 of the affected eye was 2.0–2.5 mm, which was 0.5–1.0 mm lower than the normal side. Two (5.9%) patients who underwent silicone rod suspension had equal palpebral apertures after surgery, but with the eyelid drooping again over time. For one of them, the upper eyelid height was decreased to 1.5 mm lower than the unaffected eye at the third year after surgery. For another patient, the upper eyelid height was decreased to 2.0 mm lower than the unaffected eye within 18 months. The patient underwent a secondary surgery of autogenous fascia lata suspension and had a good outcome at the end of follow-up. All the other patients (79.4%) had symmetrical palpebral aperture height and satisfactory eyelid contour of the involved eye compared with normal side.

**Figure 3:**
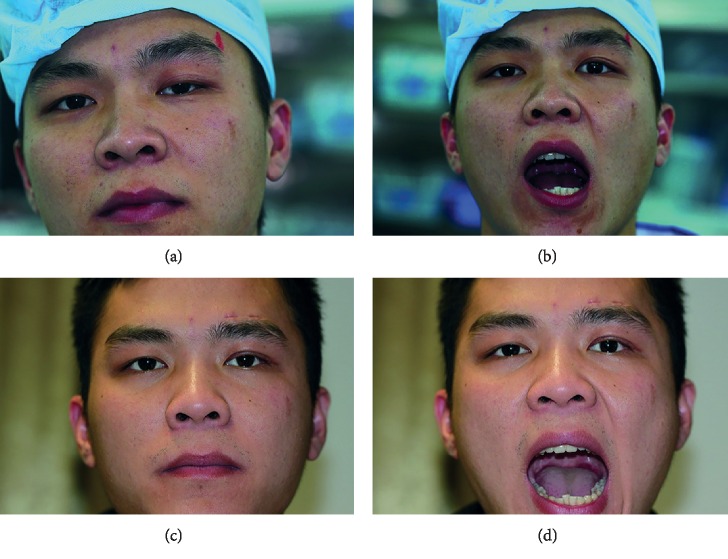
Preoperative and postoperative status of an adult patient with MGJWS of left eye. (a, b) The patient had moderate ptosis of his left eye, and the upper eyelid lifted higher than the normal side when he opened the mouth. (c, d) One month after surgery, the patient had equal palpebral apertures and symmetrical contour. Jaw-winking movement of his left eyelid was completely obliterated.

Jaw winking was resolved in all 34 patients after surgery. Twenty-eight (82.4%) patients showed complete elimination of jaw winking (Figure 3). Six patients (17.7%) had 1 mm or less residual jaw winking at the end of follow-up. In one (1/34[2.9%]) patient of the six, the synkinesis was eliminated immediately after surgery, but a mild jaw winking (1 mm) recurrent at 6 months after surgery. The potential univariate risk factors for postoperative residual jaw winking are shown in Table 2. There were significant differences in incidence of excursion distance and severity grade of jaw winking (*P*=0.002 and 0.001, respectively), as well as follow-up period (*P*=0.024 < 0.05). The mean follow-up period of patients with residual synkinesis was 53.3 months (range: 48–72 months) and 33.4 months (range: 6–72 months) for patients without residual synkinesis. Due to the significant correlation incidence between ptosis and levator function with excursion of jaw winking in the 42 patients' analysis, we included preoperative excursion grade of jaw winking, classification of ptosis, and grade of levator function into a binary multiple logistic regression analysis model, while performing further investigation of independent risk factors for residual jaw winking after surgery (Table 3). After confounding effects were controlled, preoperative severity of jaw winking showed a significant incidence of risk factor for the residual jaw winking. Patients of severe preoperative jaw-winking synkinesis had an 18.05 increased risk for postoperative residual jaw winking compared with patients of moderate preoperative jaw-winking synkinesis (*P*=0.019 < 0.05). Ptosis and levator function were not the independent risk factors for residual jaw winking after surgery (*P*=0.159 and 0.250, respectively).

Twelve patients developed lagophthalmos after surgery, but it was gradually improved over time and in most patients was disappeared after 6 months. Five (14.71%) patients still showed mild lagophthalmos (1-2 mm) at the end of follow-up. No exposure keratopathy was found in any patient with the regular use of artificial tears and lubricant. No other complications were found during the follow-up period.

## 4. Discussion

Marcus Gunn syndrome is an unusual type of congenital ptosis, presenting as blepharoptosis with synkinetic eyelid motion. In recent studies, Pearce et al. reported the incidence of MGJWS in 848 congenital ptosis cases to be 8.5% [13]. Bartkowski et al. found 13% MGP cases in congenital ptosis in 15 years' review [23]. In our study, there were 42 MGJWS patients of all congenital ptosis in 8 years, which was calculated as an incidence of 2.87%.

It is reported that MGJWS occurred more frequently on the left side and females in previous studies. Demirci et al. found 62% patients involved in left eye and 38% in right eye [12]. Digout and Awad reported a predominance of left eye of 57.6% and females of 63.6% [31]. But, in a recent study, the authors reported a high difference in frequency among male and females, in which 78% of patients were males [26]. In Pearce's review, there are 70 unilateral MG patients, of which 38 were involved right side and 32 were involved left side, and 2 were bilateral MG patients [13]. While most cases of Marcus Gunn syndrome are unilateral and sporadic, there is still a deficiency of significant statistical difference in the affected side and gender. In our study, all patients were diagnosed as unilateral Marcus Gunn jaw-winking synkinesis. The left side was affected with a higher incidence of 64.3% than the right side of 35.7%, and female patients accounted for 52.4%, while showing no significant difference with male patients (47.6%).

Surgical intervention is the main treatment for MGJWS, especially for moderate-to-severe MGJWS. Some surgeons performed levator retractor surgery or Fasanella–Servat procedure in patients with mild jaw winking and mild-to-moderate ptosis [21]. The blepharoptosis could be well corrected, and equal palpebral apertures could be achieved. However, these procedures did not obliterate the levator excursion of jaw winking and resulted in a more noticeable aberrant eyelid movement after surgery. Bowyer and Sullivan used levator aponeurosis advancement and modified conjunctivo-Müllerectomy in patients with mild MGJWS, but all these patients who underwent the procedures still had a noticeable wink after surgery [18]. For mild MGJWS, the necessity for surgical intervention should be determined both by the surgeon and the patient or parents of a pediatric patient. If the synkinesis is not considered as an unacceptable problem, clinical observation is suggested for the patients with mild jaw winking and mild ptosis. In our study, 5 patients with mild jaw winking and mild ptosis were under further observation without surgery.

It is believed that MGJWS is caused by abnormal connection between levator muscle and internal or external pterygoid. Since the specific pathogenesis of the syndrome is not yet completely clear, disabling of levator muscle so that to relieve its function in eyelid elevation is the most efficient method to eliminate the jaw-winking synkinesis. There are many surgical methods reported in previous studies, including levator muscle reserved procedures and levator muscle excised procedures. Lemagne described levator transposition to the frontalis muscle to restore some levator function [32]. This method tends to eliminate the wink but does not provide good initial correction of ptosis and often requires a subsequent procedure such as brow suspension or further tightening of the transposed levator. Bartkowski et al. performed Neuhaus method in 13 patients with marked jaw winking; of these patients, 76.9% were completely resolved and 23.1% showed residual synkinesis after surgery [23]. Bajaj et al. reported a modified technique of levator plication for the correction of Marcus Gunn jaw-winking ptosis and achieved 30% resolution (less than 1 mm residual synkinesis) of MGJWP and 70% had more than 1 mm remaining excursion with synkinesis [25]. In these methods, levator muscle or levator aponeurosis was dissected from tarsal plate and underlying tissue to weaken the function of levator muscle, but it was reserved as a suspension material to frontalis. The suspension of the upper eyelid with levator muscle might makes it more likely to retain a residual synkinesis, due to the remaining abnormal function of levator muscle and the uneliminated connection between the levator muscle and the upper eyelid.

The method of levator muscle excision followed by frontalis suspension tended to have a better outcome in synkinesis elimination. Khwarg et al. and Demirci et al. reported the resolution incidence of jaw-winking synkinesis of 97% and 85.2%, respectively, with levator muscle excision and frontalis suspension in their studies [10, 12]. Cates and Tyers also used the same procedure to Marcus Gunn patients and found that the jaw winking was well resolved in all patients; only 2 patients developed a minimal recurrent jaw winking after surgery [27]. Bowyer and Sullivan resected 1 cm of partial levator muscle above Whitnall's ligament, and the method achieved a complete elimination rate of 84.6% in moderate-to-severe MG patients [18].

The levator palpebrae superioris originates above the annulus of Zinn and transits from muscle to aponeurosis at the forming site of Whitnall's ligament. The levator aponeurosis inserts into the anterior aspect of the tarsal plate and is approximately 14–20 mm in length [33]. In most of the previous techniques, levator muscle was excised at 1–1.5 cm above Whitnall's ligament. However, persistent jaw winking after surgery has also been reported. And, there is a potential risk to damage the other superior orbital structures below levator muscle [34, 35]. In our technique, we dissected levator aponeurosis from tarsal plate and underlying tissue of eyelid upward to Whitnall's ligament and transected the levator aponeurosis under the Whitnall's ligament. Approximately, a length of 15–25 mm levator aponeurosis was excised, according to the heterogenetic anatomy of different patients. This technique could effectively release the elevatory function of levator muscle to the upper eyelid, as well as protect the deep orbital tissues such as superior rectus and superior oblique muscles below levator muscle. In our study, jaw winking was well resolved in all moderate-to-severe patients, and it completely disappeared in 85.3% patients immediately after surgery. All the surgeries were performed by an experienced oculoplastic surgeon JY. It is important to separate and excise the medial and lateral fibers of levator muscle as much as possible in order to avoid residual function of the synkinetic levator. Meanwhile, cautery of the stump margin could reduce the reattachment of levator muscle to surrounding eyelid tissues to avoid the recurrence of jaw-winking synkinesis. Of our 34 operated patients who underwent this procedure, only one patient (2.9%) developed recurrent synkinesis after operation.

We investigated the association between jaw winking and ptosis as well as levator function in this work. In previous reports, most MGJWS patients presented moderate-to-severe jaw-winking synkinesis and moderate-to-severe blepharoptosis [12, 18]; however, the potential relationship between the factors has not been well analyzed. In this study, there were 88.1% of patients presenting moderate-to-severe MGJWS, especially the moderate patients accounted for 69.1%; and 83.3% of patients presenting moderate-to-severe blepharoptosis, which were significantly different compared with patients with mild jaw winking and blepharoptosis. The interactive correlation analysis was performed in these patients and showed that ptosis and function of levator muscle had a statistically significant correlation with excursion of jaw winking. This result suggests that the more serious the jaw-winking synkinesis is, the more likely it is to be accompanied by a serious ptosis and correspondingly poor levator function. It might be related to the abnormal levator muscle fibers occurred in the involved eyelid of MGJWS patients. Lyness et al. did histological examination of the levator muscle in patients with the Marcus Gunn jaw-winking phenomenon and found that there was a loss of fibers, as well as neurogenic atrophy and aberrant reinnervation of the remaining fibers in the affected levator muscle. The aberrant fibers are relative to the synkinetic eyelid movement, and the reduction of normal muscle fibers may lead to varying degrees of dysfunction in levator muscle and result in blepharoptosis [36]. However, the distribution of abnormal fibers in affected levator muscle and the quantitative relationship between jaw-winking synkinesis of eyelid movement and the abnormal fibers were not further investigated in the study. Furthermore, we carried out statistical analysis about the residual synkinesis of surgical patients. The severity of preoperative jaw-winking synkinesis was found to be significantly correlated with the incomplete elimination of synkinetic eyelid movement after surgery. The result indicated that the more serious the jaw winking synkinesis was, the more difficult it would be to completely eliminate the synkinesis through surgery intervention. It is more prone for severe MGJWS to remain an eyelid synkinesis than moderate MGJWS. This may also be related to the amount and distribution of aberrant fibers in the affected levator muscle. The mechanism of jaw-winking synkinesis and pathological changes in eyelid tissue remain to be clarified in future, which is helpful to improve the treatment technique and prognosis for MGJWS. In our study, residual synkinesis showed significant relationship with follow-up period. It is considered to be related to the operational proficiency of the surgeon. The patients who underwent operation in the early years were more likely to suffer a postoperative residual synkinesis than patients undergoing surgery later in the study period.

In this study, the surgeon excised levator aponeurosis of the affected eye and followed by unilateral frontalis suspension of the same eye. Some surgeons suggested bilateral surgery, including bilateral levator excision and unilateral levator excision of affected eye followed by bilateral frontalis suspension. Bilateral frontalis suspension was advocated by many surgeons due to better symmetrical result compared with unilateral frontalis suspension in downgaze, because it would be easier for patients to use both sides of brow or frontalis muscle to control a symmetrical eyelid height [10, 12, 27]. However, it requires considerable understanding for patients or their parents to accept bilateral surgery as well as on the normal side. The bilateral procedure may take more complex manipulation and induce more possibility to injure potential tissues compared with unilateral surgery [37]. Of many variable frontalis suspension materials, the autogenous fascia lata and silicone rod were proved to have a satisfactory flexibility in reducing the upper eyelid lag in downgaze [38–41]. In our study, all the patients underwent unilateral operation with autogenous fascia lata or silicone rod suspension. The patients achieved satisfactory resolution of both synkinetic movement and symmetric palpebral fissure height. Although the upper eyelid height in downgaze was mildly higher than normal side in 67.7% of the patients, no patient considered it as a bothering problem that required further treatment. Mild lagophthalmos occurred in 14.7% of the patients but no exposure keratopathy was found.

## 5. Conclusions

MGJWS mostly presented as moderate-to-severe jaw-winking synkinesis of upper eyelid and accompanied with moderate-to-severe blepharoptosis. Eyelid excursion of jaw winking has a direct correlation with ptosis and dysfunction of levator muscle. Unilateral levator aponeurosis excision and frontalis suspension with autogenous fascia lata or silicone rod is an effective surgical approach in the management of unilateral MGJWS, which achieved both satisfactory symmetry outcome and resolution of eyelid movement with jaw winking. However, severe jaw winking is a risk factor for residual eyelid synkinesis after surgery. It is difficult to completely eliminate the synkinetic eyelid movement in all the severe MGJWS patients with present surgical procedures.

## Figures and Tables

**Table 1:** Numbers and proportion of patients in classification of jaw winking, blepharoptosis, and levator function.

Jaw winking	Mild (<2 mm)	Moderate (2–5 mm)	Severe (>5 mm)	Total
Blepharoptosis				
Mild (≤2 mm)	4 (80%)	3 (10.34%)	0	7/42 (16.7%)
Moderate (2–4 mm)	1(20%)	20 (68.97%)	3 (37.5%)	24/42 (57.1%)
Severe (>4 mm)	0	6 (20.69%)	5 (62.5%)	11/42 (26.2%)
P	≤0.001^*∗∗*^	0.037^*∗*^		
Levator function				
Good (>10 mm)	2(40%)	1 (3.4%)	0	3/42 (7.1%)
Fair (5–10 mm)	3(60%)	8 (27.6%)	0	11/42 (26.2%)
Poor (<5 mm)	0	20 (69.0%)	8 (100%)	28/42 (66.7%)
P	0.002^*∗∗*^	0.070		
Total	5/42 (11.9%)	29/42 (69.1%)	8/42 (19.0%)	42

^*∗*^ *P* < 0.05,^*∗∗*^*P* < 0.01.

**Table 2:** Univariate risk factors of residual jaw winking after surgery.

Clinical data	Residual jaw winking (9/34)	No residual jaw winking (25/34)	*P* value
Age (Y)	8.2 ± 6.2	11.9 ± 11.3	0.366
Gender			
Male	3	13	0.336
Female	6	12	
Affected eye			
Right	4	6	0.655
Left	5	19	
Anesthesia method			
General	8	21	0.723
Local	1	4	
Frontalis suspension material			
Autogenous fascia lata	8	22	0.943
Silicone rod	1	3	
Follow-up (month)	53.3 ± 19.1	33.4 ± 22.4	0.024^*∗*^
Excursion of jaw winking (mm)	4.67 ± 1.39	3.34 ± 0.72	0.001^*∗∗*^
Amount of ptosis (mm)	4.39 ± 1.14	3.62 ± 0.96	0.059
Excursion of LPS^†^ (mm)	3.44 ± 1.69	3.82 ± 1.64	0.564
Grade of ptosis			
Mild	0	3	0.104
Moderate	4	17	
Severe	5	5	
Levator function			
Good	0	0	0.914
Fair	2	6	
Poor	7	19	
Grade of jaw winking			
Mild	0	0	
Moderate	4	23	0.002^*∗∗*^
Severe	5	2	

^†^LPS: levator palpebrae superioris. ^*∗*^*P* < 0.05,^*∗∗*^*P* < 0.01.

**Table 3:** Independent risk factors of residual jaw winking after surgery (binary multiple logistic regression analysis).

Variable	OR	95% CI	*P* value
Jaw winking	18.05	1.81–179.96	0.014^*∗*^
Blepharoptosis	4.26	0.57–32.10	0.159
Levator function	3.94	0.38–40.66	0.025

^*∗*^ *P* < 0.05.

## Data Availability

The data used to support the findings of this study are available from the corresponding author upon request.
